# Correlation between alteration of enamel roughness and tooth color

**DOI:** 10.4317/jced.54881

**Published:** 2018-08-01

**Authors:** Waldemir-Francisco Vieira-Junior, Isabele Vieira, Glaucia-Maria-Bovi Ambrosano, Flávio-Henrique-Baggio Aguiar, Débora-Alves-Nunes-Leite Lima

**Affiliations:** 1 DDS, MSc, PhD, Professor, São Leopoldo Mandic Institute and Dental Research Center, Campinas, São Paulo, Brazil; 2 Undergraduate student, Piracicaba Dental School, University of Campinas, Piracicaba, São Paulo, Brazil; 3 MSc, PhD, Professor, Piracicaba Dental School, University of Campinas, Piracicaba, São Paulo, Brazil; 4 DDS, MSc, PhD, Professor, Piracicaba Dental School, University of Campinas, Piracicaba, São Paulo, Brazil

## Abstract

**Background:**

To establish the correlation between enamel roughness and color change of tooth.

**Material and Methods:**

Enamel/dentin blocks (5 x 5 x 3.2 mm) were serially ground with the following abrasive paper: 1200-grit, 800-grit, and 600-grit SiC papers. In the paired model, the analyses of color (L*, a*, b*, ΔE) and roughness (Ra) were performed among the sandpaper exposure. The data were subjected to ANOVA using models for repeated measures followed by the Tukey test. The Pearson correlation test was used to determine whether there was a relationship between Ra values and color results (α = 0.05).

**Results:**

The L* values decreased in accordance with the increase of Ra, with statistical difference between all the times (*p*<0.05). A correlation was found between the Ra vs. the L* values (r = -0.67; *p*<0.0001) and ∆Ra vs. ∆a* values (r = 0.29; *p* = 0.05); besides that, there was no significant correlation with b* values or significant alteration in the ∆E values (*p*>0.05).

**Conclusions:**

The alteration of enamel roughness acted on the lightness and the green-red axis of tooth color. However, there was no significant correlation between the alteration of roughness of enamel and general color change of tooth.

** Key words:**Surface properties, tooth discoloration, color.

## Introduction

The emphasis on cosmetic dentistry has increased in recent years, since the smile is one of the most important functions used for communication among people. Currently, dental aesthetics seems to be associated with tooth color, texture, position, alignment, shape, size, proportionality, and overall smile appearance (1,2). Among the morphological dental characteristics, the roughness of the surface and color are relevant properties.

Teeth are polychromatic structures composed of tissues with different optical properties, and their color is determined by the combined effects of intrinsic and extrinsic colorations (3). The intrinsic coloration of the teeth is associated with the dispersion and light absorption properties of the enamel, dentin, and pulp; however, the dentine determines the general color of the tooth (3-5). The enamel is considered a crystalline (5) tissue that, due to the arrangement of the prisms, translucency, and opalescence, confers the ability to transmit light to the underlying dentin, which features several nuances and three-dimensional aspects of color (6).

The phenomenon of observed color is the result of light scattering; illuminating light follows irregular light paths through the dental structure before it emerges at the surface of incidence and reaches the eye of the observer (7,8). Concerning that the specular reﬂection at the surface is a relevant step in the general color of an object (8), studies evaluating the role of roughness and morphology of enamel surface are necessary because changes of this nature are common in dental practice.

The changes in surface roughness are associated with accumulation of the pigments (3) and retention/accumulation of bacterial biofilm (9,10), which may impair the aesthetics of the smile. Different treatments, habits, conditions, or oral diseases can compromise the enamel surface roughness (10) such as: traumatic toothbrushing; toothbrushing with abrasive dentifrice; non-cavitated caries lesions; polishing and finishing after restorative treatment; over orthodontic bonding and debonding procedures; abrasion defects; congenital defects of structure tooth; dental bleaching; and microabrasion. Considering the situations that may alter the enamel topography and the absence of evidence, this emphasizes the relation between the increased enamel roughness and the color changes of the tooth, which is common in the daily practice of dentists. The aim of this study was to evaluate the correlation between roughness and enamel color change through a statistical correlation model. The null hypothesis tested was: 1) there is no correlation between the surface roughness of the enamel and the color of the tooth, represented by the CIE L*a*b* color system.

## Material and Methods

Sound bovine incisors teeth were stored in a 0.01% thymol solution at 4°C for 30 days until use. Enamel/dentin blocks of 5 x 5 x 3,2 mm, with 1,2 mm of enamel and 2 mm of dentin, were obtained from the middle third of the buccal surface using a low-speed, water-cooled diamond saw (Isomet, Buehler Ltd, Lake Bluff, IL, USA). The specimens were then subsequently serially ground with 600-, 800-, and 1200-grit SiC papers (Buehler Ltd) and polished with cloths and diamond spray (1, 0.5, and 0.25 µm, Buehler Ltd). All specimens were placed in an ultrasonic machine for 10 min (Marconi, Piracicaba, São Paulo, Brazil) to remove residual particles and smear layers. After obtaining a standardized enamel surface, in order to evaluate the existence of a correlation between enamel roughness and color, the blocks were submitted to a slight controlled abrasion of the surface with different SiC papers. Between each abrasion step, the color changes by the CIE L*a*b* color system (ΔE, L*, a*, b*) and roughness (Ra) using a profilometer tester were determined. All prepared specimens were stored in distilled water, which was renewed every day in order to simulate the humidity of the oral environment.

Based on a paired evaluation, the blocks were serially ground on the following abrasive grinding paper:

- Exposure to 1200-grit SiC paper for 20 s (Baseline values)

- Exposure to 800-grit SiC paper for 10 s (Intermediary values)

- Exposure to 600-grit SiC paper for 5 s (Final values)

The exposure time of each specimen in the sandpaper was chosen in accordance with the results obtained in a pilot study. Between each exposure, the block thickness was determined using a digital caliper (Mitutuyo, São Paulo, Brazil) in order to consider the role of thickness in the correlation between roughness alteration and tooth color.

The color measurements were performed at an ambient light condition (GTI MiniMatcher MM 1, GTI Graphic Technology, New York, NY, USA) in standardized daylight at different times: after 1200-grit SiC, after 800-grit SiC, and after 600-grit SiC. The color was measured using a reflectance spectrophotometer (CM 700d, Minolta, Osaka, Japan) and quantified based on the CIE L*a*b* color system, using On Color software (Konica Minolta). The L* coordinate represents the luminosity (white-black) axis, a* represents the green-red axis, and b* represents the blue-yellow axis. The spectrophotometer was initially calibrated using white and black reflectance standards in accordance with the manufacturer’s indications. Moreover, the differences in the L*, a*, and b* values between times were expressed (ΔL, Δa, and Δb) to enable all comparisons in the Pearson´s correlation, and any color change was calculated using the following equation: ΔE = [(ΔL*)2 + (Δa*)2 + (Δb*)2]1/2. The initial L* values were used to allocate specimens into the experiment aimed to reduce the initial variability, whereas the L* value is a significant parameter when making comparisons under the study design (11). Thirty specimens were initially investigated for allocating the specimens, and L* values differing 1.5% from the mean were excluded. As the study’s objective was to evaluate the effect of the enamel surface on tooth color, five samples were excluded during the experiment because they demonstrated an alteration of surface profile angle between the abrasive grinding papers, establishing a n=15.

The enamel roughness analysis (Ra) was performed using a profilometer (Surf-Corder 1700, Kosaka, Tokyo, Japan) at different times: after 1200-grit SiC, after 800-grit SiC, and after 600-grit SiC. Three different equidistant directions were measured on the surface of each specimen, with a cut-off of 0.25 mm, a reading length of 1.25 mm, and a velocity of 0.1 mm/s. The coordinate values (L*, a*, b*), roughness data (Ra), and the ∆ values of the variables (∆ = final value - initial value) were acquired in order to provide the evaluation of the Pearson´s correlation between the variables and the construction of graphs.

After exploratory analysis using the SAS software (Release 9.1, 2003, SAS Institute Inc, Cary, NC, USA), the data were subjected to ANOVA using models for repeated measures followed by the Tukey´s test. The Pearson correlation test was used to determine whether there was a relationship between roughness and color data (Δ or coordinates). The significance level was established at 5% for all analyses. The values of Ra and L*, a* and b* coordinates were directly correlated by the statistical analysis aiming to evaluate the role of enamel roughness in the color appearance of the tooth. Moreover, the values of ∆Ra and color variation (∆L*, ∆a*, ∆b*, and ∆E) were correlated due to the comparative characteristics of different times of these variables, essential for the accomplishment of the Pearson correlation test. The assessment of deltas was used to determine the role of alteration of enamel roughness in change of tooth color, such as that occurring in treatments, disorders, conditions or diseases in the oral environment.

## Results

The roughness, color, and thickness results are presented in  Table 1. The roughness analysis indicates an increase of Ra related to the increase of the sandpaper’s abrasivity, being that all groups differed statistically between them (*p* < 0.0001). The exposure to 600-grit SiC paper had the highest increase of Ra, which statistically differed from all other frames (*p* < 0.0001). Based on color results, a statistical difference in L* values among the times was found (*p* < 0.0001). The 1200-grit SiC group presented the highest L* values that were statistically different from 800-grit SiC and 600-grit SiC (*p* < 0.0001), while 600-grit SiC had already shown the smallest L* values that were statistically different from the other grits (*p* < 0.0001). For a* and b* results, the 800-grit SiC showed values that were statistically different from the 600-grit SiC or 1200-grit SiC (*p* < 0.001), which were statistically similar between them (*p* > 0.05). In relation to the thickness of specimens, there is a decrease of the thickness comparing 600-grit SiC and 1200-grit SiC (*p* < 0.001), which demonstrated statistical difference (*p* = 0.0011). The thickness values found in the 800-grit SiC group were statistically similar to the other grits (*p* > 0.05).

**Table 1:**
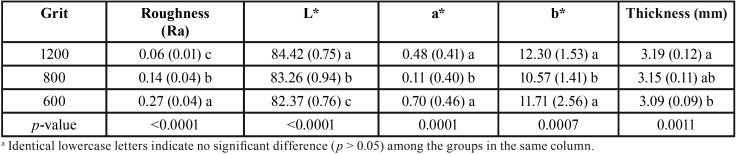
Mean (SD) for enamel roughness, L*, a*, b*, and thickness of specimens exposed serially to abrasive grinding paper (n = 15).a

The ∆E values are presented in  Table 2, where no statistical differences were found among the evaluated times in different comparisons (*p* = 0.5355), indicating that these abrasive grinding papers did not act directly on the ∆E values of the specimens.

**Table 2:**
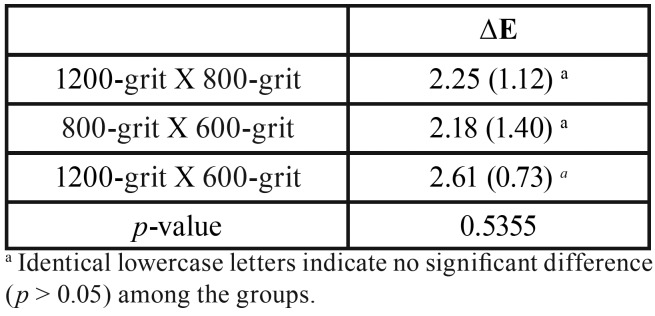
Mean (SD) for ∆E values comparing the different times of exposure (n = 15).a

**Figure 1:**
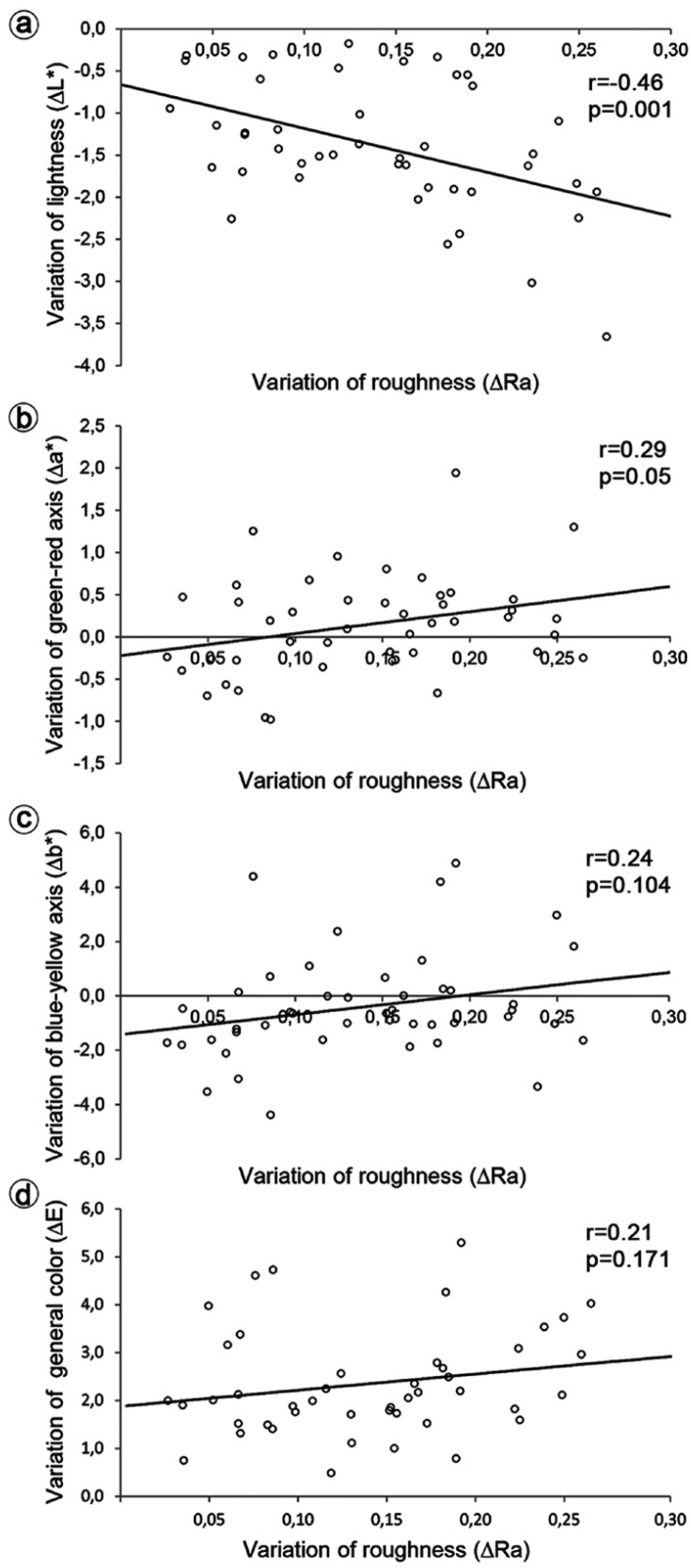
Scatter plot of ∆Ra values vs. A) ∆L* values; B) ∆a* values; C) ∆b* values, and D) ∆E values. Legend: r represents the Pearson´s correlation coefficient.

There were statistically significant correlations according to Pearson´s correlation coefficients and the *p*-value presented in  Table 3 and figure 1. The Pearson’s correlation matrix showed a significant negative correlation between roughness (Ra) and L* values (r = -0.67, *p* < 0.0001), indicating that a decrease of L* values is impacted by the increase of surface roughness. In figure 1, this correlation is again validated in the evaluation ∆Ra vs. ∆L*, which demonstrated a negative correlation (r = -0.46, *p* = 0.0013). For thickness results of table 3, the roughness values also appear to be correlated with the thickness of the specimens (r = -0.37, *p* = 0.0123). The specimens’ thickness showed a statistically positive correlation with L* values (r = 0.44, *p* = 0.0021) and a negative correlation with a* values (r = -0.35, *p* = 0.0195), so a decrease in the thickness of the block is related to a decrease in L* values and an increase in a* values. The correlation between roughness and a* or b* values was not statistically significant (*p* > 0.05), although a positive correlation between a* and b* has been found (r = 0.61, *p* < 0.0001).

**Table 3:**
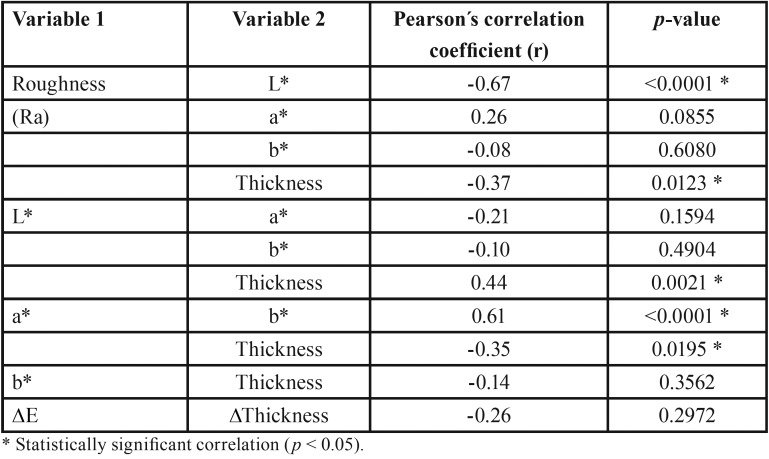
Pearson correlation analysis between the variables evaluated in the present study.

In this context, as shown in figure 1, when comparing the ∆ values, a correlation was found for ∆Ra vs. ∆a* (r = 0.21, *p* = 0.05), besides the correlation already mentioned about (∆Ra vs. ∆L*). As shown in  Table 3 and figure 1, no statistically significant correlations were found in the comparison of the following variables: Ra values vs. b* values (*p* = 0.6080); L* values vs. a* values (*p* = 0.1594); L* values vs. b* values (*p*=0.4904); b* values vs. thickness (*p* = 0.3562); ∆E values vs. ∆thickness (*p* = 0.2972); ∆E values vs. ∆Ra values (*p* = 0.1718); and ∆b* values vs. ∆Ra values (*p* = 0.1044).

## Discussion

In this study, the null hypotheses tested were partially accepted because a correlation was found between the alteration of enamel roughness values and the L* and a* values of the color spectrum; besides that, there is no significant correlation with b* values or significant alteration in the general color change represented by ∆E values. The increase of roughness was correlated with a decrease of L* values that represent the white-black axis relating to the luminosity of tooth. In this sense, the increase of roughness variation presented a correlation with the variation of a* values promoting a change in the green-red axis in the direction of red or against the green. The color appearance can be mediated by different factors: the light source, the object viewed, and the observer viewing the object (12). The focus of the present study was the evaluation of the object, the tooth, represented by the surface topographic alteration of enamel, and, for this, the color was determined in a standardized geometry of illumination and measurement.

The determination of teeth color is difficult in clinical practice by dentists, and these color changes have been scientifically measured by several authors at different methodologies (13-18). The modern approach to color can be deﬁned by value, chrome, hue, and color coordinates (19,20). The Commission Internationale de l’Eclairage (CIE) L*a*b* color scale has been used for non-self-luminous objects, and this system is widely used in aesthetic dentistry. The L* coordinate corresponds to the value or degree of lightness, ranging from 0 (black) to 100 (white); the a* coordinate indicates the redness (a > 0) or greenness (a < 0); and the b* coordinate represents the variation of yellow (b > 0) or blue (b < 0). In the present study, the highest correlation was found between roughness and L* values. This finding is clinically relevant since the L* coordinate is associated with the value of teeth that indicates the lightness of a color, and when color is determined using the Munsell system, value is determined first, followed by the aesthetics procedure, because rearrangement of the shade guide from the lightest to darkest is recommended (20,21).

The results demonstrated a correlation among roughness, L* values, a* values, and thickness. Tooth color and appearance are complex phenomena, and, for some events that abrade or erode the enamel to the extent of inducing loss of structure in depth, the color changes appear to intensify. A previous study (4) suggested that tooth color is mainly determined by dentin; however, the present study shows that alteration of the enamel surface is able to change the light scattering or light reflected by the enamel. A considerable fraction of the light entering the tooth is probably lost because it emerges at the outside surface, whereas it is suggested that the tooth shade could be regulated by the size of the hydroxyapatite enamel crystals (22). The difference between the b* or a* values was determined only in the roughness of 1200- and 800- groups, suggesting that a slight alteration of enamel texture affects the color distribution of the tooth. The values of a* do not represent the natural chrome of the tooth and are possibly related to the pigments incorporated in the dental structure, and the results suggest that a diffused reflection also acted in the dispersion and absorption of light in the visible light spectrum, especially in longer wavelength and lower frequency regions, as in the perception of red-based tones.

However, there is no statistical differences for a* and b* values when the specimens were exposed to 600-grit SiC, possibly due to the small decrease in thickness that allowed reestablishment at the degree of light absorption of the dental substrates, particularly in the underlying dentin. Conversely, determination of the color spectrum could have been interfered with by the alteration in surface reflectance and the light reflected from the enamel surface, which was confirmed from the L* results of all groups. Thus, it is possible to hypothesize that the grater loss of tooth structure can expose the optical characteristics of dentin, whereas the translucency of enamel increases in the inverse proportion to thickness and in direct proportion to the wavelength (23).

Despite the fact that the investigation of the role of roughness on tooth color is clinically essential, the results of Pearson´s correlation presented here are important for future study models that aim for the evaluation of these variables, thus contributing new insights. The alteration of enamel texture promoted by abrasive grinding paper could act in the orientation of enamel rods/prisms and alter the chromatic properties of the tooth. In this way, different clinical procedures may result in the change of enamel topography such as: microcracks and enamel fractures; scratches and abrasions caused by forcibly removing brackets; traumatic toothbrushing or abrasive toothpastes; and dental bleaching (10,24-27). In relation to enamel debonding procedures, a previous study (24) showed that tooth color variables are affected and that the differences observed exceed the threshold for clinical detection. Additionally, the previous investigation (27) calculated the relationship between the physical surface properties of bleached enamel, represented by microhardness or roughness alteration and the color properties using a multivariate canonical correlation analysis, which has shown that the variation in the tooth color explained 21% of the variation in the physical surface variables. In the present study, the correlation coefficient was higher because, unlike the hydrogen peroxide that acts through enamel and dentin, in the present study the abrasive changes occurred on the enamel surface.

Concerning the clinical procedures, when the light interacts with the object, several processes can occur, including reflection, transmission, absorption, scattering, and fluorescence, which can be altered by the surface characteristics of the object (12,28). In the present study, there was color alteration for all change of roughness, including for small topographic variations of the enamel. However, the present investigation did not demonstrate a correlation between the alteration of roughness and ∆E values; nevertheless, during the comparison, all groups ranged from 2 to 3 ∆E units that are visually perceptible considering the difference of ∆E values, proposed by Alghazali and others (29), being 1.9 ∆E units for the assessment of perceptibility and 4.2 ∆E units for the clinical acceptability of color differences. Thus, considering the relevance of tooth color on smile attractiveness and appearance (1), clinical techniques need improvement and development in order to promote a safe treatment, especially relating to recovering the surface texture of damaged enamel.

## Conclusions

The change in the surface roughness of the enamel affected the lightness and the green-red axis of tooth color, correlating respectively with the L* and a* values. However, there was no significant correlation between the alteration of roughness and the general change of tooth color, represented by ∆E values.
